# The paradoxical impact of drought on West Nile virus risk: insights from long-term ecological data

**DOI:** 10.1098/rspb.2025.1365

**Published:** 2025-09-03

**Authors:** Samantha Sambado, Terrell J. Sipin, Zoe Rennie, Ashley Larsen, James Cunningham, Amy Quandt, Dan Sousa, Andrew J. MacDonald

**Affiliations:** ^1^ University of California Santa Barbara, Santa Barbara, CA, USA; ^2^ Stanford University, Stanford, CA, USA; ^3^ San Diego State University, San Diego, CA, USA

**Keywords:** climate variability, bird host community, vector-borne disease, rural–urban landscapes, panel regression models, *Culex* spp.

## Abstract

Mosquito-borne diseases are deeply embedded within ecological communities, with environmental changes—particularly climate change—shaping their dynamics. Increasingly intense droughts across the globe have profound implications for the transmission of these diseases, as drought conditions can alter mosquito breeding habitats, host-seeking behaviours and mosquito–host contact rates. To quantify the effect of drought on disease transmission, we use West Nile virus as a model system and leverage a robust mosquito and virus dataset consisting of over 500 000 trap nights collected from 2010 to 2023, spanning a historic drought period followed by atmospheric rivers. We pair this surveillance dataset with a novel modelling approach that incorporates monthly changes in bird host community competence, along with drought conditions, to estimate the effect of drought severity on West Nile virus risk using panel regression models. Our results show that while drought decreases mosquito abundances, it paradoxically increases West Nile virus infection rates. This counterintuitive pattern probably stems from reduced water availability, which concentrates mosquitoes and pathogen-amplifying bird hosts around limited water sources, thereby increasing disease transmission risk. However, the magnitude of the effect depends critically on mosquito species, suggesting species-specific behavioural traits are key to understanding the effect of drought on mosquito-borne disease risk across real landscapes.

## 1. Background

Climate change and extreme climate events are major threats to human health [1,2]. Human health risks, such as vector-borne diseases, are particularly sensitive to changes in climate and are an increasing health threat in North America [3,4]. Ectothermic vectors that rely on water for breeding, such as mosquitoes, are sensitive to changes in temperature and the availability of standing water [5,6]. Drought (drier-than-normal conditions) can reduce the availability of standing water, thereby potentially affecting mosquito development rates and breeding success [5,7]. Many diseases spread by mosquitoes involve various species of vectors and hosts, which may respond differently to drought even when they coexist within the same community [8,9]. To identify the effects of drought on mosquito-borne disease risk, it is essential to differentiate between species-specific responses, enabling more nuanced vector control strategies and public health messaging.

Drought is an important driver of ecosystem dynamics [10,11], which affects mosquito populations that rely on water to complete their life cycle [7,12,13]. Reduced precipitation can lead to diminished habitats for egg laying, thereby decreasing mosquito abundances [5,7,12]. However, reduced precipitation can decrease the total area of standing water, potentially concentrating mosquitoes and hosts that can become infected and transmit infections back to mosquitoes [14]. Increased contact rates between mosquitoes and important hosts of pathogens can increase pathogen transmission rates [14–16]. In drought-affected regions, natural waterways such as rivers can provide essential water resources across the landscape [8]. However, rivers that rely on snowmelt can experience substantial fluctuations depending on the timing and rate of snowmelt, which are influenced by drought conditions [12,17–19]. Parsing apart the potentially conflicting impacts of drought on two critical metrics for mosquito-borne disease risk (mosquito abundance and infection rates) will be essential as droughts are projected to become more frequent and severe [7,11].

West Nile virus (WNV) has been the most common mosquito-borne disease in North America since its introduction to the United States in 1999 [4,20]. Given that WNV relies on multiple factors such as mosquito abundances, the presence of disease-carrying hosts such as birds, and human contact rates, a mechanistic understanding of drought effects is crucial for predicting the risk of human exposure to mosquito-borne disease [21]. WNV is transmitted by many *Culex* spp. mosquitoes and primarily maintained by bird hosts [22]. Certain bird species, known as ‘competent hosts’, can acquire and maintain the virus, serving as infectious blood meals for mosquitoes. In humans, infections are mostly asymptomatic (approx. 80% of known cases), but they can be severe, resulting in neuroinvasive disease in about approximately 1% of known cases [4]. Over the past 20 years, California has accounted for 15% of all reported human cases of WNV in the United States, with the Central Valley reporting incidence rates that are roughly twice the state average [4,20,23]. This is thought to be due to extensive agricultural production requiring irrigation, which enhances mosquito breeding habitat [6]. Additionally, agricultural regions often have higher concentrations of low-income communities facing challenges like poor housing, limited healthcare access and inadequate water infrastructure [23–26]. For example, in Kern County, the 2020 median household income was $67 660, with nearly 20% of the population living below the poverty line [27]. These conditions heighten disease risk and can prevent timely medical care, likely contributing to under-reporting of cases. In this context, environmental analyses provide a critical tool for identifying gaps in surveillance and targeting public health interventions—underscoring the environmental justice dimensions of mosquito-borne disease risk.

Situated in the southern part of California’s Central Valley, Kern County has an average of less than 153 mm of precipitation per year and has experienced multiple historic droughts over the past two decades (electronic supplementary material, figure S1) [10,28]. During that time, Kern County has also experienced persistent, and in some years, relatively high rates of WNV activity in both mosquitoes and humans (table 1). The county’s land use is predominantly agricultural, centred around the major city of Bakersfield with a population of approximately 400 000 [27]. Of the 2.3 million acres of classified farmland, roughly 30% are irrigated [30]. With contrasting rural agricultural and urban settings, there are two significant vectors of WNV: *Culex tarsalis* and *Culex quinquefasciatus*, considered the primary rural and urban vectors of WNV, respectively [17]. The rural vector prefers breeding in larger bodies of water, such as seasonal wetlands, and experiences higher population numbers following an increase of water on the landscape. In contrast, the urban mosquito is more likely to breed in small containers and artificial water sources, making it more susceptible to being flushed out by heavy rainfall or large water pulses [8,12]. The disease potential of these vectors also differs, where the rural mosquito can transmit higher pathogen loads but typically comes in less contact with humans compared to the urban mosquito, which exploits peridomestic environments that provide more opportunities to feed on humans [17,31]. However, rural mosquitoes are more likely to encounter vulnerable populations such as agricultural workers who may not have access to public health messaging in their native language or access to healthcare [24,25]. Our study area, which has experienced recent and extreme variations in drought conditions, along with consistent, longitudinal mosquito surveillance activities, presents a compelling opportunity to explore the effects of drought on mosquito-borne disease risk.

**Table 1.** Summary of annual climate conditions, mosquito abundance and infection rates, and human WNV incidence in Kern County. Total cumulative precipitation (precip., mm) from gridMET [29] was calculated and averaged for each clusterID-water year (November through March). Mosquito metrics include the mean number of mosquitoes per trap night and the mean WNV minimum infection rate (MIR) (±standard deviation) during peak mosquito months (April through October), reported separately by *Culex* species. Human WNV incidence data reported per 100 000 people.^a^

year	climate	mosquito				human
	precip.	count ± s.d. per trap night		WNV MIR ± s.d. per trap night		disease incidence per 100 000 people^a^
		*C. tarsalis*	*C. quinq*.	*C. tarsalis*	*C. quinq*.	
2010	153	7.7 ± 25	15.0 ± 38	2.6 ± 8	4.8 ± 11	1.65
2011	259	10.7 ± 26	8.0 ± 16	3.3 ± 12	4.0 ± 12	1.98
2012	129	6.9 ± 39	10.0 ± 24	4.1 ± 10	5.1 ± 11	2.75
2013	82	16.4 ± 126	9.5 ± 20	4.5 ± 9	4.3 ± 10	2.86
2014	84	11.0 ± 47	11.9 ± 36	6.1 ± 13	4.0 ± 11	1.21
2015	128	3.4 ± 10	13.5 ± 58	4.9 ± 9	5.6 ± 11	1.21
2016	163	9.5 ± 55	8.4 ± 25	4.8 ± 10	2.6 ± 8	1.87
2017	221	14.2 ± 54	6.8 ± 14	3.6 ± 10	7.7 ± 14	3.30
2018	108	8.0 ± 51	5.8 ± 17	1.1 ± 4	1.2 ± 5	1.43
2019	192	11.6 ± 44	7.6 ± 20	2.6 ± 8	3.4 ± 8	3.08
2020	192	12.0 ± 108	9.0 ± 22	1.6 ± 6	1.7 ± 6	0.88
2021	82	5.3 ± 19	4.4 ± 15	7.3 ± 13	4.1 ± 11	0.88
2022	133	9.3 ± 72	5.7 ± 14	3.5 ± 10	3.8 ± 9	2.42
2023	320	68.4 ± 155	10.6 ± 25	1.3 ± 6	2.9 ± 10	1.76

^a^ Number of reported human WNV disease cases from ArboNET were divided by the Kern County 2020 census population (909 235), then multiplied by 100 000.

In this study, we investigate the effects of drought severity on mosquito abundance and WNV infection rates in Kern County, California, between 2010 and 2023. We aim to build upon prior small-scale empirical studies that suggest drought differentially impacts two important mosquito-borne disease risk metrics—mosquito abundances and WNV infection rates [12,17,32]—here using a novel data-driven approach. We use drought data and field surveillance data from 278 trap stations that were deployed over 500 000 trap nights resulting in 3.6 million mosquitoes to estimate changes in mosquito abundance and WNV infection rates through time and across variable drought conditions to address the following questions:

(1) How do *C. tarsalis* and *C. quinquefasciatus* abundances respond to drought severity?
(2) How does WNV infection prevalence in *C. tarsalis* and *C. quinquefasciatus* respond to drought severity?

## 2. Methods

### (a) Mosquito and WNV surveillance data

**Figure 1.**
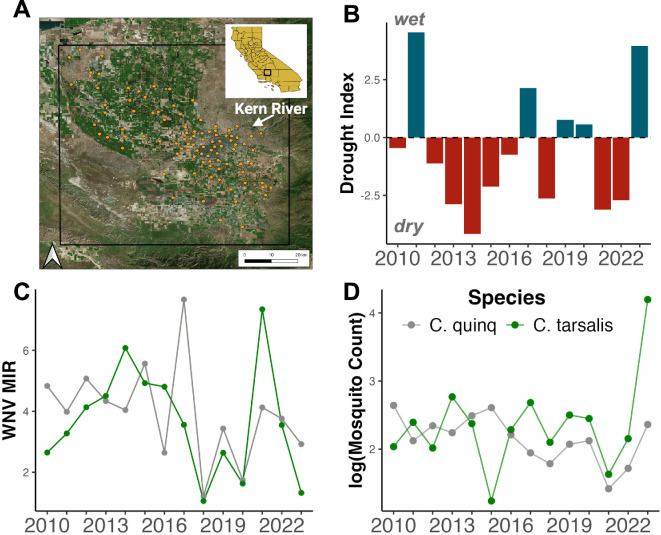
Study system. (*a*) ClusterID locations centred around Bakersfield, CA. (*b*) Yearly mean PDSI drought index. (*c*) Yearly mean WNV minimum infection rate (MIR). (*d*) Log_10_ of yearly mean mosquito count per clusterID. In plots (*c*) and (*d*), mosquito species are colour-coded: grey for *C. quinquefasciatus* (urban vector) and green for *C. tarsalis* (rural vector).

The Kern Mosquito and Vector Control District conducts regular mosquito surveillance with standardized trapping throughout the year, increasing their efforts during the summer months when mosquito activity peaks [8]. The number of mosquitoes is reported per trap night and includes information on mosquito species, life stage and sex. Mosquitoes from the same trap, trap night and species are aggregated into pools and screened for WNV infection. For this study, we use data from the peak mosquito activity months of April through October during 2010−2023 and only on *C. tarsalis* and *C. quinquefasciatus* adult female mosquitoes, the primary vectors of WNV in Kern County, and their respective WNV infection rates (table 1; figure 1) [17].

To produce more consistent local estimates of abundance and WNV infection through time at an individual station, as well as account for spatial autocorrelation and non-independence of mosquito abundances in highly spatially aggregated trap stations, we spatially clustered nearby trap stations following the approach of MacDonald *et al*. [33]. We cluster trap stations within approximately 1500 m radius based on the daily dispersal rate of *Culex* spp. in the San Joaquin Valley [34]. Clusters of trap stations were created using a complete-linkage hierarchical clustering method with a tree height cutoff of approximately 3000 m using the ‘hclust’ function in R (R Core Team 2024). Each cluster contains distinct trap stations, so clusters of trap stations do not contain duplicate surveillance data, resulting in 96 unique clusters (electronic supplementary material, figure, S2). After clustering trap stations into 96 unique clusters (referred to as clusterID), we calculate monthly averages of (i) the number of mosquitoes per trap night and (ii) the WNV minimum infection rate (MIR) for each cluster. The MIR was calculated with the following equation: MIR=([number of positive  pools/number of mosquitos  tested]× 1000).

### (b) Environmental and ecological covariates

**Figure 2.**
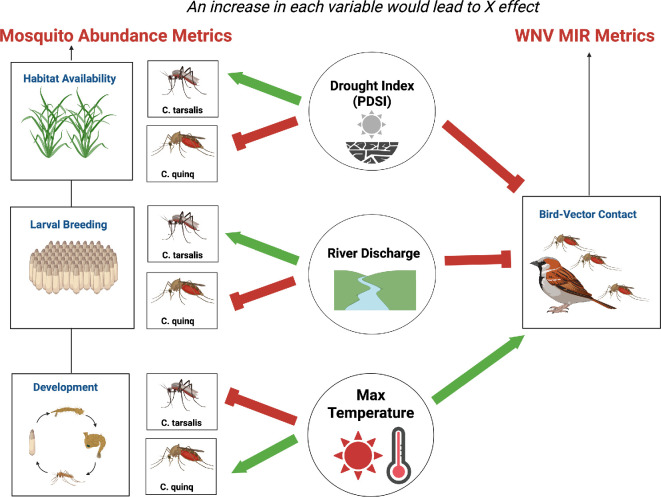
Hypothesized relationship between drought measurements, adult *Culex* spp. mosquito abundances and West Nile virus minimum infection rates (WNV MIR). Changes in a drought measurement (circle) can either negatively (red) or positively (green) affect mosquito abundance or bird–mosquito contact rates, influencing WNV dynamics. The Palmer Drought Severity Index (PDSI), which ranges from 4 (wet) to −4 (dry), indicates wetter conditions with higher values. Created in BioRender (https://BioRender.com/1f9vtl4).

Mosquito abundance and WNV risk are influenced by various factors, including the physiological development of individual mosquitoes, the transmission dynamics of WNV, the quality of larval breeding sources and the availability of habitats for adult mosquitoes and their blood hosts [6,7,21]. Some environmental drivers, such as river discharge, may lead to rapid, short-term changes by creating temporary larval breeding sites, while others, such as prolonged drought conditions, can cause more gradual, long-term shifts in habitat availability and ecosystem suitability (figure 2). To isolate the impact of drought on mosquito abundance and WNV infection rates, we use the following set of covariates in our analysis: Palmer Drought Severity Index (PDSI), Kern River discharge rates and bird community competence index. Data are summarized by clusterID within the 1500 m radius buffer around trap station cluster centroids. By spatially clustering buffers using their centroids rather than individual trap stations, we can calculate average environmental values even when some stations are missing in a given month-year. This approach smooths fine-scale variability while preserving ecological relevance and generates a dataset well-suited for panel models. Covariates in our models are standardized.

**Figure 3.**
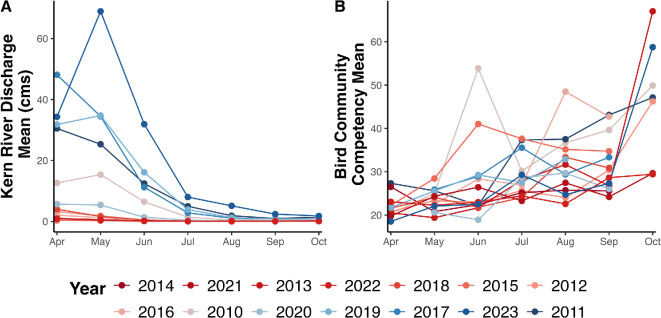
Monthly and yearly variation of environmental variables affecting (*a*) larval breeding water availability and (*b*) likelihood of mosquitoes taking an infectious bird blood meal. Colours represent the mean Palmer Drought Severity Index (PDSI) for each year, from the driest (−4.2 in 2014) to the wettest (4.5 in 2011).

To quantify drought conditions throughout our study period, we use the standardized PDSI from gridMET, which estimates the relative soil moisture conditions every 5 days. A PDSI value of >4 represents very wet conditions, while a PDSI value of <−4 represents extreme drought [29]. We average the PDSI per time step (month-year) and clusterID (figure 3). We hypothesize that a positive PDSI value represents a landscape more suitable for larval breeding, with sufficient vegetation shelter for adults and their blood meal hosts, compared to a negative PDSI value. We also include a month-lag PDSI value to represent drought conditions in the prior month, assuming that a wetter prior month would be more conducive to mosquito breeding and influence adult mosquito abundance in the current month, in contrast to a drier prior month.

To capture potential effects of water input into the system that are not captured by drought conditions (i.e. PDSI index), we included the monthly average discharge rate of the Kern River (cms) that is the same for all clusterIDs per month-year [19]. The discharge rate data were sourced from the United States Geological Survey National Water Information System (USGS NWIS) for station SF Kern R NR Onyx, CA (11189500) (latitude: 35.7374516, longitude: −118.173689) [35]. The Kern River watershed is a dynamic source of water (figure 3) that is dependent on the Sierra Nevada snowpack, with water released from Lake Isabella tailored to meet local municipal and agricultural needs [12,17]. During years of above-normal rainfall, the Kern River flowing through Bakersfield causes intentional flooding of ponds to recharge aquifers and directs excess flow into Lake Buena Vista, located east of Bakersfield [12,17]. Previous studies have found a correlation between increased discharge rates of the Kern River and *C. tarsalis* abundance [18]. We hypothesize that during years with relatively high Kern River discharge rates, there will be more standing water in the surrounding areas of Bakersfield, which will increase available breeding habitat for *C. tarsalis*, increasing their abundances. While the Kern River itself may not serve as a direct breeding habitat for *C. quinquefasciatus*, increased river discharge can influence nearby small artificial containers and storm drains—common urban breeding sites for this mosquito—by flushing out larvae and thus reducing *C. quinquefasciatus* populations in areas where the river flows through Bakersfield.

In addition to climate covariates, birds are generally understood to be key ecological drivers of WNV transmission dynamics, serving as amplification hosts of vector-borne diseases. Thus, it is important to incorporate the abundance of birds in the study area and consider their species-specific relative competencies as potential hosts of WNV, as not all birds that migrate through or are residents of Kern County are competent hosts for WNV [22]. Building upon MacDonald *et al*., we created a bird community competence index that we averaged by month-year for each clusterID (figure 3). Specifically, the relative abundances of 38 bird species inhabiting the Central Valley were modelled using *Best Practices for Using eBird Data* adopted from the Cornell Ornithology Laboratory to replicate eBird status and trends products [36] (electronic supplementary material, table S1). Further details can be found in the electronic supplementary material, text S1. We hypothesize that months with higher bird community competence resulted in a higher probability of a mosquito taking an infectious blood meal, resulting in a higher WNV MIR.

### (c) Isolating the mosquito-borne disease risk and drought relationships

Randomized controlled experiments are the gold standard for inferring causal effects in ecological communities [37,38]. However, in observational studies involving complex ecological systems, it is often infeasible to randomly assign treatment due to resource limitations or ethical concerns [37]. Here, we use a within-estimator panel model (also known as a ‘fixed effects’ model in econometrics) to approximate randomized experiments in observational data settings [37,38]. These models enable us to account for unit-specific, time-invariant characteristics, as well as shared time shocks or seasonality, which allow us to better isolate and more accurately estimate the impact of time-varying variables on our outcomes of interest.

Mosquito-borne disease risk is impacted by multiple abiotic and biotic factors; however, our focus is the impact of drought on mosquito abundance and WNV infection rates. To parse drought from other relevant factors, we use a within-estimator model with time (i.e. month) and individual (i.e. clusterID) fixed effects in addition to key time-varying biotic and abiotic covariates hypothesized to influence our outcome variables of interest. Key time-varying variables such as Kern River discharge rate and bird community competence influence mosquito abundance and mosquito–bird contact rates, which can impact WNV transmission rates. Our goal is to account for or remove, through differencing, all variables that are correlated with our covariate of interest (i.e. drought severity; PDSI) and our outcomes, as any such variable that remains in the error term would bias our estimation. Variables that drive the outcome but are not correlated with drought would increase noise but would not bias our coefficient estimates.

Our final mosquito abundance model predicts the number of mosquitoes per trap night in clusterID *i* and month *t*. To accommodate zero values in the abundance data, we added 1 prior to a ${log}_{10}$-transformation. Our outcome is (log(${Abundance}_{it}+1$)), which we model as a function of the drought severity from the previous month (${PDSI}_{it-1}$) and is written as


(2.1) $$log\left({Abundance}_{it}+1\right)=\alpha_{i}+\beta_{1}\left({PDSI}_{it-1}\right)+\beta_{2}\left({River}_{it}\right)+c_{i}+\gamma_{t}+\varepsilon_{it}$$

We control for average Kern River discharge rates of the contemporaneous month and include the fixed-effect dummy variables clusterID ($c_{i}$) and month ($\gamma_{t}$). We include the terms $\alpha_{i}$ for clusterID intercept and $\varepsilon_{it}$ as the random error. We run separate models for each focal species, *C. tarsalis* and *C. quinquefasciatus*.

Our final mosquito infection model predicts the WNV MIR in clusterID *i* and month *t*. To accommodate zero values in the MIR data, we added 1 prior to a ${log}_{10}$ transformation. Our outcome is (log(${MIR}_{it}+1$)), which we model as a function of drought severity index from the previous month (${PDSI}_{it-1}$) and is written as


(2.2) $$log\left({MIR}_{it}+1\right)=\alpha_{i}+\beta_{1}\left({PDSI}_{it-1}\right)+\beta_{2}\left({River}_{it}\right)+\beta_{3}\left({Bird}_{it}\right)c_{i}+\gamma_{t}+\varepsilon_{it}$$

We additionally control for average Kern River discharge rates of the contemporaneous month and the average bird host community competence of the contemporaneous month and include the fixed-effect dummy variables clusterID $\left(c_{i}\right)$ and month $\gamma_{t}$. We include the terms 𝛼_𝑖_ for clusterID intercept and 𝜀_𝑖𝑡_ as the random error. We run separate models for each focal species, C. *tarsalis* and C. *quinquefasciatus*, infection rates.

We estimated the within-estimator panel models using the feols function from the ‘fixest’ package [39]. For our final models, we calculate Conley standard errors to account for spatial autocorrelation of the standard errors (see electronic supplementary material, text S2, for further model justifications).

All statistical analyses were performed using R software v. 4.4.1 (R Studio Core Team 2024). Data cleaning and visualizations were conducted with the ‘tidyverse’, ‘ggplot2’, ‘modelsummary’ packages, respectively [40–42]. Code for figure making and the panel model analysis can be found at GitHub [43].

## 3. Results

A total of 2 353 573 C. *tarsalis* (per trap night mean = 14.0 ± 82.1) and 1 321 196 C. *quinquefasciatus* (per trap night mean = 9.3 ± 35.0) adult female mosquitoes were captured from April through October between 2010 and 2023 in Kern County (table 1). Of those mosquitoes captured and tested, *C. tarsalis* and *C. quinquefasciatus* have a mean WNV MIR of 3.2 (±9.5) and 4.1 (±10.5), respectively.

### (a) *Culex* spp**.** mosquito abundances are impacted by drought severity

**Figure 4.**
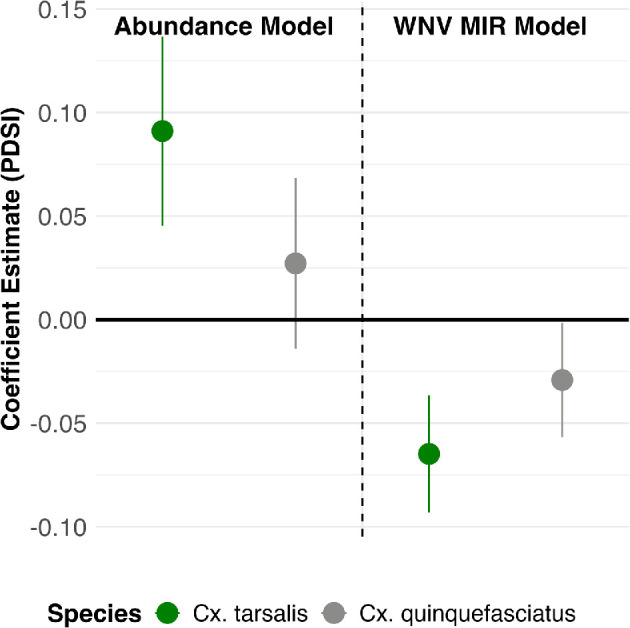
Coefficient estimates and 95% confidence intervals for panel models of the log_10_-transformed mosquito abundances and WNV minimum infection rates (WNV MIR), by species. Conley standard errors were used to account for spatial correlation. Note that an increase in PDSI indicates increased wetness.

We found a significant effect of drought severity on the abundances of *C. tarsalis*, the rural mosquito (figure 4; electronic supplementary material, tables S2 and S3, figures S3–S5). A higher value of the drought index means wetter conditions; thus, our results can be interpreted as a 1 s.d. increase in wet conditions leads to a 9% increase in *C. tarsalis* abundance (beta = 0.09 ± 0.023). We did not find a significant effect for *C. quinquefasciatus* abundance (beta = 0.027 ± 0.021), but it was positively trending similarly to *C. tarsalis* (table 2). As expected, the control covariate for Kern River discharge rate was positive for both mosquito species but had a larger impact on *C. tarsalis* (beta = 0.35 ± 0.047) compared to *C. quinquefasciatus* (beta = 0.058 ± 0.014). This suggests that our panel models are effectively isolating the effect of drought on mosquito abundances, independent of the water pulses into the system not captured by drought metrics.

**Table 2.** Results for the preferred model specification best fit for the abundance and WNV minimum infection rate (MIR) models for both species of mosquitoes, *C. tarsalis* and *C. quinquefasciatus*, with the fixed effects of clusterID *i* and month *t*. Conley standard errors were used to account for spatial correlation of the standard errors. All covariates are the monthly average standardized. We bolded *p*-values that corresponded to our main covariate of interest (PDSI_*t*−1_).

		**e**stimate	Conley s.e.	*t*-value	***p*‐**value
abundance models	*C. tarsalis*				
	PDSI_*t*−1_	0.09	0.02	3.91	**<0.001**
	river discharge_*t*_	0.36	0.05	7.54	<0.001
	*C. quinquefasciatus*				
	PDSI _*t*−1_	0.03	0.02	1.29	**0.20**
	River discharge _*t*_	0.06	0.01	4.03	<0.001
WNV MIR models	*C. tarsalis*				
	PDSI _*t*−1_	−0.064	0.01	−4.51	**<0.001**
	River discharge _*t*_	−0.032	0.01	−6.44	<0.001
	Bird community _*t*_	0.092	0.04	2.27	0.02
	*C. quinquefasciatus*				
	PDSI _*t*−1_	−0.029	0.01	−2.07	**0.04**
	River discharge _*t*_	−0.036	0.01	−5.99	<0.001
	Bird community _*t*_	0.034	0.03	1.31	0.02

### (b) WNV infection rates are impacted by drought severity

We found a significant effect of drought on the West Nile infection rate of both mosquito species (figure 4; electronic supplementary material, tables S4–S5, figures S6–S8). However, the magnitude of the effect differed between mosquito species. A 1 s.d. increase in wet conditions of the prior month leads to a 6% and 3% decrease in WNV MIR infection of *C. tarsalis* (beta = −0.064 ± 0.014) and *C. quinquefasciatus* (beta = −0.029 ± 0.014), respectively (table 2). The control covariates for Kern River discharge rate and bird community competence were trending in the expected directions (negative for Kern River, positive for bird competence) further suggesting our within-estimator panel models are isolating the effect of drought on WNV MIRs, independent of pulses of water into the system not captured by drought metrics and controlling for changes in bird host community competence.

## 4. Discussion

Climate change is driving an increase in both the frequency and severity of droughts [10,11]. In Mediterranean climates like California, this is resulting in abrupt shifts between periods of intense droughts and severe atmospheric river and precipitation events, profoundly impacting entire ecological communities [10,28]. Drought exerts both direct and indirect influences on the risk of mosquito-borne diseases [5,7]. We find that drought is reducing mosquito abundances while concurrently increasing WNV infection rates. However, the extent to which drought affects the abundance of two key WNV vectors, *C. tarsalis* and *C. quinquefasciatus*, varies. We hypothesize that this variation is due to their distinct ecological niches, which directly influence their breeding preferences and responses to drought conditions. Drought may indirectly influence WNV infection rates by reducing bodies of water on the landscape, potentially intensifying mosquito–bird contact rates and thereby contributing to increased WNV infection rates [7,17]. Here, we present evidence of contrasting effects of drought on two critical metrics of mosquito-borne disease, but the effects differ from one vector species to another, suggesting within the same period, human health risks will vary from rural to urban settings. Our findings underscore the importance of species-specific responses to drought, carrying significant implications for public health messaging and resource allocations for control measures.

The risk of mosquito-borne diseases depends on both mosquito abundance and infection rates—two outcomes that are affected by drought but respond in distinct ways. Our findings revealed that after a dry period in the previous month (indicated by a lower PDSI index), mosquito abundances decreased while WNV infection rates increased. These findings align with our hypotheses and support previous studies [7,12,14,16,17,32], which suggest that drought reduces available mosquito breeding habitats, thereby lowering population numbers. Drought reduces not only suitable breeding habitat but also the overall area of standing water on a landscape, which may lead to spatial aggregation of mosquitoes and competent bird hosts, potentially increasing contact rates and enhancing WNV transmission [7,17]. As expected, we found a positive relationship between WNV infection rates and bird community competence. However, we did not measure host aggregation directly, so this remains a hypothesized mechanism. The average magnitude of drought’s effect on WNV infection rates was smaller than that of our primary biological variable—bird community competence index—suggesting that other ecological factors may also contribute to observed patterns of WNV infection rates [21,44,45]. An alternative hypothesis is that drought conditions reduce the recruitment of nulliparous females by diminishing available aquatic habitat, leading to lower vector abundance and shifting the age structure in mosquito populations to older mosquitoes, which may increase infection prevalence. Although temperature is a key driver of both mosquito population dynamics and WNV transmission, it was not a significant predictor in our initial models. This result is not unexpected, given that our mosquito trap locations are relatively clustered within a region characterized by homogenous summer temperatures, which may limit the variability captured across study units—particularly when month-of-year dummy variables are included in the models (electronic supplementary material, figure S9). As California experiences more dramatic shifts in drought severity, mosquito-borne disease risk may similarly be increasingly amplified and dampened in response. However, future research should investigate how prolonged severe droughts affect transmission potential and determine whether there are thresholds in the response of mosquitoes and bird hosts that might decouple mosquito-borne disease risk from drought conditions.

The impact of drought on mosquito abundance differs between the two key vector species in Kern County, California. Our findings show that drought has a more pronounced effect on the rural vector, *C. tarsalis*, compared to the urban vector, *C. quinquefasciatus*. This suggests that understanding species-specific habitat and behavioural traits is crucial for understanding the impact of drought on mosquito ecology [8,12,32]. The rural mosquito, *C. tarsalis*, which is commonly found in agricultural areas, appears to be more sensitive to drought fluctuations, possibly because it has less refugia than the urban mosquito during unfavourable drought conditions [12,17]. While *C. quinquefasciatus*, the urban mosquito, was negatively affected by drought, its abundances exhibited less variation in response to fluctuating drought conditions than the rural mosquito [17]. This resilience may stem from the mosquito’s ability to seek refuge and breed in human-made containers such as swimming pools, ornamental plants and urban trash [44]. Another aspect of drought that may differentially impact mosquito species is the eutrophication of aquatic habitats, which occurs more frequently under drought conditions. *Culex tarsalis* is less tolerant of hypereutrophic, polluted water conditions compared to *C. quinquefasciatus*, which may further explain why increasing drought has a greater effect on *C. tarsalis* populations. As expected, our mosquito abundance models showed that Kern River discharge had a stronger effect on the rural vector (beta = 0.36 ± 0.05) than on the urban vector (beta = 0.06 ± 0.01). This difference likely reflects breeding preferences: increased river discharge benefits rural mosquitoes that rely on large water bodies (e.g. flooded aquifers, seasonal wetlands), whereas urban mosquitoes—typically breeding in small containers—are less influenced. However, even minimal increases in water availability (i.e. positive PDSI value) may still boost their populations, as some water on the landscape is better than none [12,18,19].

While our study identifies contrasting effects of drought on WNV risk that vary across vector species, it is essential to consider these results in the context of the Central Valley. In both rural and urban settings, humans modify water availability through irrigation and landscaping practices. Even during drought years, substantial federal and local funding supports the extensive agricultural irrigation networks that can supplement natural breeding habitats. Regions in California, such as Sacramento, Bakersfield and Coachella Valley, are endemic for WNV but differ in their water management practices, which can complicate predicting WNV risk in response to drought [8,17]. Sacramento and Bakersfield benefit from natural waterways (i.e. Sacramento and Kern rivers), especially the Sacramento-San Joaquin River Delta, which is less prone to drying out during drought years [8]. In contrast, Coachella Valley relies more heavily on human-driven irrigation rather than natural waterways, potentially altering the impact of drought on mosquito abundances and WNV risk depending on human behaviour. Without the natural supplement of water, the effect of drought on West Nile risk may be greater by increasing infection rates in mosquitoes, or if natural water bodies are robust to drought, then mosquito populations will remain stable. Another factor that could influence our mosquito abundance results is the application of pesticides by vector control agencies and private individuals. Some preliminary data suggest that due to the consistency of pesticide application by Kern Mosquito and Vector Control District staff, there would be little effect on our general results that encompass 14 years of surveillance data (electronic supplementary material, figure S10). Another aspect that warrants further investigation is the impact of land cover type on our results. With the within-estimator panel models, we aimed to remove variation that is unique to individual trap stations [37,38], but there could be further investigation due to future land use change as a result of projected human population growth in California’s Central Valley or the Sustainable Groundwater Management Act, which may lead to the fallowing of large areas currently in agricultural production [46].

Evaluating mosquito-borne disease risk in response to drought must also account for areas where human populations are likely to encounter mosquitoes [23–25]. In our study system, we observe varying magnitudes of response to drought between rural and urban mosquitoes, highlighting important considerations for public health messaging. During wet years, the rural mosquito *C. tarsalis* tends to experience a greater increase in abundance compared to the urban mosquito *C. quinquefasciatus*. This finding suggests that agricultural workers, who may have limited access to public health information or inequitable access to healthcare, are potentially at a higher risk of WNV compared to individuals primarily spending time in urban areas [17,25,26]. In dry years, when rural areas pose lower risk, mosquito mitigation efforts may be better allocated to urban settings, particularly in areas near the Kern River or residential pools, which could serve as more consistent water refuges for mosquito populations when residential water use (e.g. landscaping) may be reduced [44,45]. Although our study analysed mosquito abundance and infection rates separately to better understand their distinct responses to drought conditions, a vector index (which incorporates both abundance and infection rate) or calculating maximum likelihood rather than MIR could serve as valuable and more accurate metrics for assessing disease risk (electronic supplementary material, table S6) [47]. The differential responses of rural and urban mosquitoes to drought underscore the need for targeted public health strategies that account for varying risk levels across different environments and human populations.

## 5. Conclusion

Our study investigated the effect of drought severity on a complex vector-borne disease with a multi-vector system. We found that 1 month lagged drought conditions can exert contrasting effects on mosquito abundance, infection rates and ultimately mosquito-borne disease risk. Increased drought severity negatively affects mosquito abundances directly. In contrast, West Nile infection rates are positively influenced by increased drought severity, likely due to reduced water availability concentrating mosquito vectors and bird hosts, increasing vector–host contact rates and thereby potentially increasing transmission risk. Importantly, the effects of drought vary between vector species, *C. tarsalis* (rural) and *C. quinquefasciatus* (urban), underscoring the need for nuanced public health messaging and mitigation strategies as drought conditions fluctuate in California.

## Data Availability

Data and code to recreate figures and analyses are available via GitHub [43] and are stored at the Dryad Digital Repository [48]. Details on data sources used for the analyses can be found in the electronic supplementary material, table S7. A preprint of this work can be found at bioRxiv [49]. Supplementary material is available online [50].

## References

[1] Ebi KL, Ogden NH, Semenza JC, Woodward A. 2017 Detecting and attributing health burdens to climate change. Environ. Health Perspect. **125**. (10.1289/ehp1509) PMC5783629 28796635

[2] Baker RE *et al*. 2022 Infectious disease in an era of global change. Nat. Rev. Microbiol. **20**, 193–205. (10.1038/s41579-021-00639-z) 34646006 PMC8513385

[3] Swei A, Couper LI, Coffey LL, Kapan D, Bennett S. 2020 Patterns, drivers, and challenges of vector-borne disease emergence. Vector Borne Zoonotic Dis. **20**, 159–170. (10.1089/vbz.2018.2432) 31800374 PMC7640753

[4] McDonald E, Mathis S, Martin SW, Staples JE, Fischer M, Lindsey NP. 2021 Surveillance for west nile virus disease — United States, 2009–2018. MMWR Surveill. Summ. **70**, 1–15. (10.15585/mmwr.ss7001a1) PMC7949089 33661868

[5] Chase JM, Knight TM. 2003 Drought‐induced mosquito outbreaks in wetlands. Ecol. Lett. **6**, 1017–1024. (10.1046/j.1461-0248.2003.00533.x)

[6] Kovach TJ, Kilpatrick AM. 2018 Increased human incidence of west nile virus disease near rice fields in california but not in Southern United States. Am. J. Trop. Med. Hyg. **99**, 222–228. (10.4269/ajtmh.18-0120) 29714160 PMC6085780

[7] Paull SH, Horton DE, Ashfaq M, Rastogi D, Kramer LD, Diffenbaugh NS, Kilpatrick AM. 2017 Drought and immunity determine the intensity of West Nile virus epidemics and climate change impacts. Proc. R. Soc. B **284**, 20162078. (10.1098/rspb.2016.2078) PMC5310598 28179512

[8] Barker CM, Eldridge BF, Reisen WK. 2010 Seasonal Abundance of Culex tarsalis and Culex pipiens Complex Mosquitoes (Diptera: Culicidae) in California. J. Med. Entomol. **47**, 759–768. (10.1093/jmedent/47.5.759) 20939368 PMC2965637

[9] Campbell R, Thiemann TC, Lemenager D, Reisen WK. 2013 Host-Selection Patterns of Culex tarsalis (Diptera: Culicidae) Determine the Spatial Heterogeneity of West Nile Virus Enzootic Activity in Northern California. J. Med. Entomol. **50**, 1303–1309. (10.1603/me13089) 24843936

[10] Prugh LR, Deguines N, Grinath JB, Suding KN, Bean WT, Stafford R, Brashares JS. 2018 Ecological winners and losers of extreme drought in California. Nat. Clim. Chang. **8**, 819–824. (10.1038/s41558-018-0255-1)

[11] Slette IJ, Post AK, Awad M, Even T, Punzalan A, Williams S, Smith MD, Knapp AK. 2019 How ecologists define drought, and why we should do better. Glob. Chang. Biol. **25**, 3193–3200. (10.1111/gcb.14747) 31276260

[12] Reisen WK, Cayan D, Tyree M, Barker CM, Eldridge B, Dettinger M. 2008 Impact of climate variation on mosquito abundance in California. J. Vector Ecol. **33**, 89–98. (10.3376/1081-1710(2008)33[89:iocvom]2.0.co;2) 18697311

[13] Harrigan RJ, Thomassen HA, Buermann W, Smith TB. 2014 A continental risk assessment of West Nile virus under climate change. Glob. Chang. Biol. **20**, 2417–2425. (10.1111/gcb.12534) 24574161

[14] Shaman J, Day JF, Stieglitz M. 2005 Drought-Induced Amplification and Epidemic Transmission of West Nile Virus in Southern Florida. J. Med. Entomol. **42**, 134–141. (10.1093/jmedent/42.2.134) 15799522

[15] Kilpatrick AM, Kramer LD, Jones MJ, Marra PP, Daszak P. 2006 West Nile virus epidemics in North America are driven by shifts in mosquito feeding behavior. PLoS Biol. **4**, e82. (10.1371/journal.pbio.0040082) 16494532 PMC1382011

[16] Shaman J, Day JF, Stieglitz M. 2002 Drought-Induced Amplification of Saint Louis encephalitis virus , Florida. Emerg. Infect. Dis. **8**, 575–580. (10.3201/eid0806.010417) 12023912 PMC2738489

[17] Reisen WK, Carroll BD, Takahashi R, Fang Y, Garcia S, Martinez VM, Quiring R. 2009 Repeated West Nile Virus Epidemic Transmission in Kern County, California, 2004–2007. J. Med. Entomol. **46**, 139–157. (10.1603/033.046.0118) 19198528 PMC2729460

[18] Wegbreit J, Reisen WK. 2000 Relationships among weather, mosquito abundance, and encephalitis virus activity in California: Kern County 1990-98. J. Am. Mosq. Control Assoc **16**, 22–27. 10757487

[19] Shutt DP *et al*. 2022 A Process-based Model with Temperature, Water, and Lab-derived Data Improves Predictions of Daily Culex pipiens/restuans Mosquito Density. J. Med. Entomol (ed. H Gaff), **59**, 1947–1959. (10.1093/jme/tjac127) 36203397 PMC9667726

[20] Snyder RE, Feiszli T, Foss L, Messenger S, Fang Y, Barker CM. 2020 West Nile virus in California, 2003–2018: A persistent threat. PLoS Neglected Trop. Dis **18**, e0008841. (10.1371/journal.pntd.0008841) PMC7710070 33206634

[21] Kala AK, Tiwari C, Mikler AR, Atkinson SF. 2017 A comparison of least squares regression and geographically weighted regression modeling of West Nile virus risk based on environmental parameters. PeerJ **5**, e3070. (10.7717/peerj.3070) 28367364 PMC5372833

[22] Kilpatrick AM, LaDeau SL, Marra PP. 2007 Ecology of West Nile Virus Transmission and its Impact on Birds in the Western Hemisphere. Auk **124**, 1121–1136. (10.1093/auk/124.4.1121)

[23] Danforth ME, Fischer M, Snyder RE, Lindsey NP, Martin SW, Kramer VL. 2021 Characterizing areas with increased burden of west nile virus disease in California, 2009–2018. Vector Borne Zoonotic Dis. **21**, 620–627. (10.1089/vbz.2021.0014) 34077676 PMC8380797

[24] Harrigan RJ, Thomassen HA, Buermann W, Cummings RF, Kahn ME, Smith TB. 2010 Economic conditions predict prevalence of west nile virus. PLoS One **5**, e15437. (10.1371/journal.pone.0015437) 21103053 PMC2980475

[25] Hernandez E, Torres R, Joyce AL. 2019 Environmental and Sociological Factors Associated with the Incidence of West Nile Virus Cases in the Northern San Joaquin Valley of California, 2011–2015. Vector Borne Zoonotic Dis. **19**, 851–858. (10.1089/vbz.2019.2437) 31211639 PMC6818473

[26] Bayles BR, George MF, Christofferson RC. 2024 Long‐term trends and spatial patterns of West Nile Virus emergence in California, 2004–2021. Zoonoses Public Health **71**, 258–266. (10.1111/zph.13106) 38110854

[27] United States Census Bureau. 2025 Bakersfield city, California. See https://data.census.gov/profile/Bakersfield_city,_California?g=160XX00US0603526.

[28] Swain DL, Langenbrunner B, Neelin JD, Hall A. 2018 Increasing precipitation volatility in twenty-first-century California. Nat. Clim. Chang. **8**, 427–433. (10.1038/s41558-018-0140-y)

[29] Abatzoglou JT. 2013 Development of gridded surface meteorological data for ecological applications and modelling. Int. J. Climatol. **33**, 121–131. (10.1002/joc.3413)

[30] United States Department of Agriculture National Agricultural Statistics Service. 2025 County profile: Kern County, California. See https://www.nass.usda.gov/Publications/AgCensus/2022/Online_Resources/County_Profiles/California/cp06029.pdf.

[31] Goddard LB, Roth AE, Reisen WK, Scott TW. 2002 Vector competence of california mosquitoes for west nile virus. Emerg. Infect. Dis. **8**, 1385–1391. (10.3201/eid0812.020536) 12498652 PMC2738502

[32] Reisen WK, Fang Y, Martinez VM. 2006 Effects of temperature on the transmission of west nile virus by Culex tarsalis (Diptera: Culicidae). J. Med. Entomol. **43**, 309–317. (10.1093/jmedent/43.2.309) 16619616

[33] MacDonald AJ, Hyon DW, Sambado S, Ring K, Boser A. 2024 Remote sensing of temperature-dependent mosquito and viral traits predicts field surveillance-based disease risk. Ecology10.1002/ecy.4420 PMC11534503 39319755

[34] Reisen WK, Milby MM, Meyer RP. 1992 Population Dynamics of Adult Culex Mosquitoes (Diptera: Culicidae) Along the Kern River, Kern County, California, in 1990. J. Med. Entomol. **29**, 531–543. (10.1093/jmedent/29.3.531) 1625303

[35] USGS NWIS. 2025 Monitoring location SF Kern R NR Onyx CA USGS 11189500. See https://waterdata.usgs.gov/monitoring-location/11189500/#parameterCode=00065&period=P7D&showMedian=false.

[36] Fink D. 2020 eBird Status and Trends. Cornell Laboratory of Ornithology. (10.2173/ebirdst.2019)

[37] Larsen AE, Meng K, Kendall BE. 2019 Causal analysis in control–impact ecological studies with observational data. Methods Ecol. Evol. **10**, 924–934. (10.1111/2041-210x.13190)

[38] Dee LE *et al*. 2023 Clarifying the effect of biodiversity on productivity in natural ecosystems with longitudinal data and methods for causal inference. Nat. Commun. **14**, 2607. (10.1038/s41467-023-37194-5) 37147282 PMC10163230

[39] Bergé L. 2019 fixest: fast fixed-effects estimation. See https://CRAN.R-project.org/package=fixest.

[40] Wickham H. 2016 Ggplot2: elegant graphics for data analysis, p. 1, 2nd edn. Cham, Switzerland: Springer.

[41] Wickham H *et al*. 2019 Welcome to the Tidyverse. J. Open Source Softw. **4**, 1686. (10.21105/joss.01686)

[42] Arel-Bundock V. 2022 modelsummary: data and model summaries in. R. J. Stat. Softw **103**, i01. (10.18637/jss.v103.i01)

[43] Sambado S. 2025 Code to recreate data summaries and analyses. See https://github.com/sbsambado/wnv_drought_kern.

[44] Reisen WK, Takahashi RM, Carroll BD, Quiring R. 2009 Delinquent mortgages, neglected swimming pools, and West Nile virus, California. Emerg. Infect. Dis. **15**, 508b–5509. (10.3201/eid1503.081611) 18976560 PMC2630753

[45] Bhattachan A, Skaff NK, Irish AM, Vimal S, Remais JV, Lettenmaier DP. 2021 Outdoor residential water use restrictions during recent drought suppressed disease vector abundance in Southern California. Environ. Sci. Technol. **55**, 478–487. (10.1021/acs.est.0c05857) 33322894 PMC9426289

[46] Public Policy Institute of California. 2025 California’s Central Valley. See https://www.ppic.org/wp-content/uploads/content/pubs/jtf/JTF_CentralValleyJTF.pdf.

[47] Moore TC, Tang X, Brown HE. 2025 Assessing the relationship between entomological surveillance indices and west nile virus transmission, United States: systematic review. Vector Borne Zoonotic Dis. **25**, 317–328. (10.1089/vbz.2024.0072) 39943921

[48] Sambado S. 2025 Data from: The paradoxical impact of drought on West Nile virus risk: insights from long-term ecological data. Dryad Data Repository. (10.5061/dryad.b8gtht7qn) PMC12404801 40897323

[49] Sambado S, Sipin TJ, Rennie Z, Larsen A, Cunningham J, Quandt A, Sousa D, MacDonald AJ. The paradoxical impact of drought on West Nile virus risk: insights from long-term ecological data. bioRxiv. (10.1101/2025.01.21.634155) PMC12404801 40897323

[50] Sambado S, Sipin TJ, Rennie Z, Larsen A, Cunningham J, Quandt A, Sousa D, MacDonald AJ. 2025 Supplementary materal from: The paradoxical impact of drought on West Nile virus risk: insights from long-term ecological data. Figshare. (10.6084/m9.figshare.c.7987125) PMC12404801 40897323

